# Determination of Tyrosine Kinase Inhibitors via Capillary Electrophoresis with Tandem Mass Spectrometry and Online Stacking Preconcentration

**DOI:** 10.3390/ph16020186

**Published:** 2023-01-25

**Authors:** Jan Petr

**Affiliations:** Department of Analytical Chemistry, Faculty of Science, Palacký University Olomouc, 17. listopadu 12, 77146 Olomouc, Czech Republic; jan.petr@upol.cz

**Keywords:** capillary electrophoresis, mass spectrometry, online preconcentration, stacking, tyrosine kinase inhibitors

## Abstract

Capillary electrophoresis connected with tandem mass spectrometry was employed for the development of a method for determination of various tyrosine kinase inhibitors in plasma samples. A stacking online preconcentration with a 120 cm-long capillary was used for the determination of bosutinib, dasatinib, canertinib, and erlotinib at physiologically relevant concentrations. The optimization included both capillary electrophoresis and mass spectrometry steps. Under optimal conditions, 50 mM formic acid pH 2.5, an injection time of 120 s, and an optimized mass spectrometry set-up (as sheath liquid composition 75:24.9:0.1 (*v*/*v*) methanol, water, formic acid, and appropriate conditions for ion transitions), LODs in a range of 3.9–23.0 nmol·L^−1^ were observed. The method was validated in terms of linearity, limit of detection, limit of quantification, repeatability of migration times and peak area, and recovery using plasma as a matrix for analytes. The results showed that this method has great promise for use in many analytical tasks, e.g., therapeutic drug monitoring.

## 1. Introduction

Tyrosine kinase inhibitors (TKIs) are a family of small molecules or peptides with the ability to inhibit either cytosolic or receptor tyrosine kinases. Inhibition by this class of agents occurs through different types of actions, where the direct competition for ATP binding to tyrosine kinase is often described [1,2]. Generally, tyrosine kinases have the function of catalyzing the transfer of a phosphoryl group from a nucleoside triphosphate donor to the hydroxyl group of tyrosine residues on protein substrates and then triggering the activation of downstream signaling cascades [3]. Abnormal activation of tyrosine kinases due to mutations, translocations, or amplifications is implicated in tumorigenesis, progression, invasion, and metastasis of malignancies [4]. As a result, tyrosine kinases have emerged as major targets for drug discovery. In 2001, the FDA approved imatinib for the treatment of chronic myeloid leukemia. However, the majority of TKIs are currently not in clinical use. The exceptions are the first-used imatinib, erlotinib, and dasatinib, which show promise as “targeted” therapeutics in the treatment of various cancers as well as leukemia [1,2].

Therapeutic drug monitoring (TDM) is the clinical practice of measuring a drug’s concentration in blood or plasma, or in other biological fluids that can be linked to blood drug concentrations. The measured drug concentration is then used to adjust a drug dosing regimen by targeting a predefined concentration or exposure interval, called a therapeutic range [5]. The WHO has issued specific guidelines on how a drug should be monitored due to TDM’s clinical importance, which is defined as individualizing a drug’s dose by keeping a drug’s concentrations in the plasma or blood within a target range to act as a guide for healthcare staff [6]. Such guidelines deal with large interpatient pharmacokinetic variability, adverse effects, therapeutic concentration-related effects, undefined therapeutic concentration ranges, and difficult-to-manage desired therapeutic effects. The WHO report also stressed certain criteria, such as an increased drug concentration in the blood, being related to increased efficacy and/or toxicity in the organism, the difficulty in monitoring a target drug’s pharmacological effects, and drug concentration-related adverse effects [7]. Likewise, the report suggested a list of a pharmacological groups requiring monitoring, i.e., antibiotics (aminoglycosides and glycopeptides), anticonvulsants (e.g., valproic acid, phenobarbital, carbamazepine), cytotoxic drugs (e.g., metotrexate), antiarrhythmics (e.g., digoxin), and immunosuppressants (e.g., cyclosporine), which are indispensable drugs for the treatment of a myriad of diseases in current clinical practice [8].

Different techniques have been necessarily employed in TDM due to the nature of the investigated drugs to be quantified. Some of the most common techniques are high-performance liquid chromatography (HPLC) and its connection with mass spectrometry (HPLC-MS), gas chromatography–mass spectrometry (GC–MS), and immunoassays. The chromatographic techniques are the most robust and specific reference techniques; however, these methods need trained personnel, involve long sample processing times, and require costly reagents. Moreover, a sample is processed in the chromatograph each time (unlike other techniques that usually do not need expensive reagents after the validation of the technique) and requires a specialized laboratory for processing [6,9]. In contrast, immunoassays have been of great use as these techniques operate in less time than HPLC and/or GC-MS (chromatographic techniques require samples and mobile-phase preparations, extractions and/or filtrations, derivatizations, and continuous control of the equipment for correct operation). However, these techniques require trained personnel and a clinical laboratory with the necessary equipment and reagents. Such techniques include radioimmunoassays, enzyme-linked immunosorbent assays (known as ELISA), and fluorescence polarization immunoassays, which have been used for quantifying antibiotics as well as anticancer/antineoplastic, antiarrhythmic, and biological drugs [6,10].

Last but not least, the capillary electrophoresis (CE) technique represents an alternative to HPLC. CE has a unique separation mechanism, speed, efficiency, and versatility. CE separation depends on the different migration of solutes in an electric field. CE is performed in narrow-bore capillaries filled with a background electrolyte (BGE). The driving forces in CE are electrophoretic migration and electro-osmotic flow (EOF). Compared with other techniques, the instrumentation of CE is simple and consists of electrodes, sample-introduction systems, a capillary, a power supply, a detector, and a liquid-handling system. Detection can be achieved with online (diode-array spectrophotometric, spectrofluorimetric, and electrochemical) or external detectors (mass spectrometer, MS) [11,12,13]. CE has been noted as a “green” technique as it “consumes” an ultralow amount of chemicals and samples. Usually, nanoliter volumes of samples are analyzed (microliters are necessary for injection from commercial instruments). In the case of chemicals, typically only a few milliliters of BGE is needed. Hence, the use of substances potentially harmful to the environment, e.g., organic solvents, is limited, especially in comparison with HPLC [14]. Moreover, since CE suffers from less-sensitive UV detection, so-called online sample-concentration techniques have been developed in the past. These include four basic approaches: stacking, transient isotachophoresis, dynamic pH junction, and sweeping. The use of such techniques can improve LOD values from tens to millions, especially when the electrokinetic injection of samples is employed. Compared to “traditional” offline sample pretreatment steps, such as liquid–liquid extraction or solid-phase extraction, online sample preconcentration techniques are user-friendly, as most enrichment processes are performed in the separation capillary. In general, the composition of zones in the capillary is “programmed” to focus analytes of interest. The simplest mechanism (stacking) is based on the analyte velocity change (e.g., slowing down) in a two-discontinuous-solutions system (e.g., at the boundary formed by diluted BGE and non-diluted BGE) [15,16,17,18].

As noted, TDM is considered a very useful tool in helping clinicians with individual dose adjustment. Many studies have highlighted the clinical benefit of TDM for the first tyrosine kinase inhibitor—imatinib [19,20,21]. Like many other TKIs, imatinib exhibits large interpatient pharmacokinetic variability with more than ten-fold differences in drug plasma concentration, which sometimes results in therapeutic failures [22,23]. This variation has been linked to multiple genetic factors but may also be influenced by other physiological and environmental factors, such as drug–drug/food interactions and patient adherence [24]. Previous studies have shown that about 95% of imatinib is bound to albumins and α-1-acid glycoproteins in plasma. The large variability in the concentration of the latter results in inconsistent concentrations of unbound and active imatinib [25]. Clinical data have also shown that the plasma concentration of imatinib is directly linked to therapeutic success; for example, in the case of chronic myeloid leukemia, the concentration needs to be higher than 1000 ng·mL^−1^, while for GISTs, the minimum concentration is 1100 ng·mL^−1^. Current literature data recommend that the therapeutic dose should not exceed 3000 ng·mL^−1^; however, a systematic analysis of available patient data, where side effects were reported, appears to suggest that a much lower value (closer to 1500 ng·mL^−1^) should be used [22].

Given the research discussed above, it is of great interest to develop new tools to measure plasma concentrations of different TKIs. Several methods have already been developed and published, mainly those connecting the use of liquid chromatography with mass spectrometry, as described previously [26,27,28,29,30,31,32]. For example, Merienne et al. presented a UPLC-MS/MS method for determination of 17 TKIs in one run using C18 column and LOD in 0.1 ng·mL^−1^ levels [30]. Koller et al. developed a new clean-up procedure for analysis of 11 TKIs with Poroshell C18 column and similar LOD levels [31]. Further, supercritical fluid chromatography with MS was used for quantitation of 11 TKIs on a DIOL column with a gradient program [32].

The use of capillary electrophoresis, a technique for analysis of TKIs with a different separation principle based on the migration of ions in an electric field, has also been described in the literature. For example, Horská et al. [33] separated seven TKIs in 7 min using phosphate buffer pH 2.75. Rodriguez et al. [34] developed a non-aqueous CE method for analysis of imatinib with its metabolite and two analgesics using a background electrolyte containing ammonium acetate and acetic acid in methanol. Gonzales et al. [35] compared analysis of dasatinib via UHPLC and CE, both with UV detection. An LOD more than one-hundred-times better was obtained by UHPLC. These papers only described the use of low-sensitivity UV detection, as the optical path for UV detection is extremely low in CE. This problem can be overcome by using different type of detection, such as mass spectrometry (which also offers identification of the compounds of interest), or by using offline or online preconcentration techniques. Here, Rodriguez et al. [36] achieved a 70 ng·mL^−1^ LOD for sunitinib using CE connected to TOF-MS. Forough et al. [37] used nanocomposite-based electromembrane extraction followed by field-amplified sample injection online preconcentration in CE with UV detection, resulting in a determination of imatinib with an LOD of 6.24 ng·mL^−1^. Nanomaterials, especially multi-walled carbon nanotubes, were also used for dispersive solid-phase extraction prior to CE-UV of dabrafenib and trametinib in serum samples (with LODs from 8 ng·mL^−1^) [38]. The Perrin group published a set of papers [39,40,41] dealing with salting-out procedures for determination of TKIs in plasma samples with LODs higher than 16 ng·mL^−1^. Acetonitrile (ACN) was used to precipitate proteins from plasma samples followed by the addition of NaCl to the ACN–plasma mixture to introduce two-phase separation. Next, the high ACN content allowed for stacking of the analytes in CE. Finally, in 2020, Zhao et al. [42] used CE-MS with a field-amplified sample stacking method for the determination of imatinib and its metabolites with an LOD of 0.2 ng·mL^−1^, which is fully comparable with those obtained by UPLC-MS/MS. 

**Figure 1:**
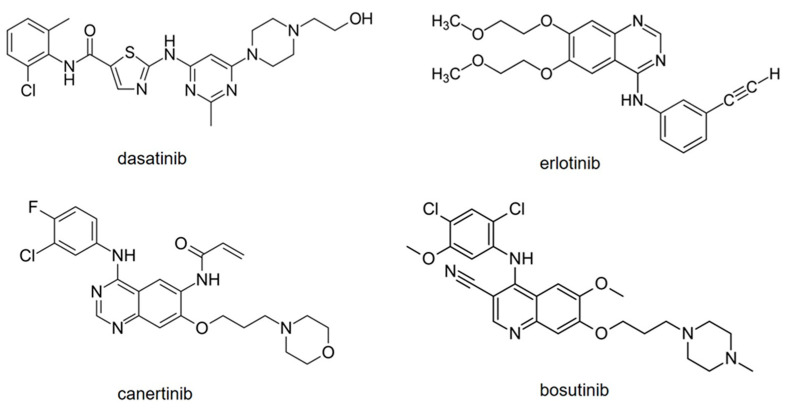
Structures of model TKIs.

Given the above, CE-MS seems to be a good alternative to HPLC-MS or UHPLC-MS for TKIs. Moreover, in contrast to HPLC (and UHPLC), CE is considered a green (environment-friendly) technique, as it uses smaller amounts of solvents and samples. Considering this, the aim of the current work was to develop a CE-MS/MS method for the determination of multiple TKIs, dasatinib, erlotinib, canertinib, and bosutinib (Figure 1), using an online preconcentration strategy. These four TKIs were chosen because of their use in clinical praxis and their different structural features. In addition, they had not been previously analyzed by CE-MS.

## 2. Results

### 2.1. Method Development

In our previous work [33], the model TKIs were separated by CZE with UV detection under acidic conditions with non-volatile phosphate buffer pH 2.75. The LODs were incomparable with the levels of these TKIs in plasma samples. However, the right pH adjustment was necessary to obtain separation. Hence, first, the effect of pH was studied using 200 mM, 100 mM, 50 mM, and 10 mM formic acid (pH 2.2, 2.4, 2.5, and 2.9, respectively) and 100 mM, 50 mM, and 10 mM acetic acid (pH 2.9, 3.0, and 3.4, respectively) as background electrolytes (BGEs). The decrease in pH led to an increase in the duration of analysis from 3 min (pH 3.4) to more than 60 min (pH 2.2). This was likely due to differences in the electroosmotic flow and sucking of MS. The best separation (the highest resolution between all the peaks) was achieved at pH 2.5. Hence, 50 mM formic acid was used in further experiments.

**Figure 2:**
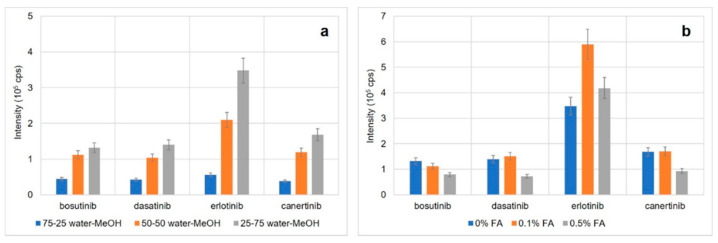
Optimization of the sheath liquid composition. (**a**) Optimization of water–methanol ratio, (**b**) effect of formic acid content; error bars represent SD values (n = 3).

Next, MS conditions were optimized. First, the effect of sheath liquid composition on TKI signals as well as background noise was studied. Initially, water–methanol mixtures at ratios of 25:75, 50:50, and 75:25 (*v*/*v*) were evaluated (without the addition of formic acid). The TKI signals were the highest for the 25:75 (water–methanol, *v*/*v*) ratio, Figure 2a; the same results were observed for the signal/noise ratio. Then, the effect of formic acid presence (0%, 0.1%, 0.5%; *v*/*v*) was analyzed (75% methanol, 25% or 24.9% or 24.5% water; *v*/*v*). The addition of 0.1% (*v*/*v*) had a dramatic effect on the erlotinib signal while the other TKIs had similar signals to those without any formic acid (Figure 2b). The higher concentration (0.5%; *v*/*v*) did not have any positive impact on the signal intensity. Similar effects were observed for the signal/noise ratio. Hence, the addition of 0.1% (*v*/*v*) formic acid was used for further optimization. Additional parameters included MS operational parameters, such as electrospray voltage (3.0–4.5 kV), drying gas temperature (200–300 °C), nebulizing gas flow rate (5–13 L min^−1^), nebulizing gas pressure (10–20 psi), and sheath liquid flow rate (0.4–1.0 μL·min^−1^). The highest TKI signals were observed at an electrospray voltage of 4.0 kV, drying gas temperature 250 °C, nebulizing gas flow rate 5 L·min^−1^, nebulizing gas pressure 15 psi, and sheath liquid flow rate 0.6 μL·min^−1^.

To obtain the correct single reaction monitoring (SRM) transitions for both determination and identification, all the TKIs were fragmented by increasing the collision energy from 0 eV to 50 eV. The SRM transitions used for the quantitation and identification are listed in Table 1. They also correspond with previously published literature [30,43]. 

### 2.2. Stacking Preconcentration

To decrease the LOD values, sample stacking preconcentration was applied. This technique is based on an extended injection of analytes in a diluted (low-conductivity) electrolyte to the background electrolyte (higher conductivity). The differences in conductivities, reflected in differences in the electric field strengths, lead to the slowdown of analytes at the boundary between the sample and electrolyte plugs and their preconcentration [44,45]. In this work, a methanol–BGE mixture (90:10, *v*/*v*) was used as the TKI background to ensure some minimal conductivity of the sample zone when using an extended injection. Next, the effect of injection time of TKIs was studied in a range of 5 to 120 s. A linear increase in the TKI peak area was observed up to 60 s. LOD values were roughly estimated to be about 1 × 10^−7^ mol·L^−1^. This is quite high given practical (therapeutic drug monitoring) applications. In addition, the resolution between the TKIs rapidly decreased (with an 85 cm-long capillary) and there was not enough time for stacking. Roughly, a length of about 6 cm, representing about 7% of the capillary length, is injected within 60 s. Theoretically, about 40 cm of the capillary length is needed for the separation of the TKIs (the mobilities of TKIs are: bosutinib 29.7, canertinib 27.5, dasatinib 22.7, and erlotinib 19.6, all 10^−9^ m^2^V^−1^s^−1^). The motion of TKIs in the rest of the capillary is likely caused by the suction of MS. 

**Figure 3:**
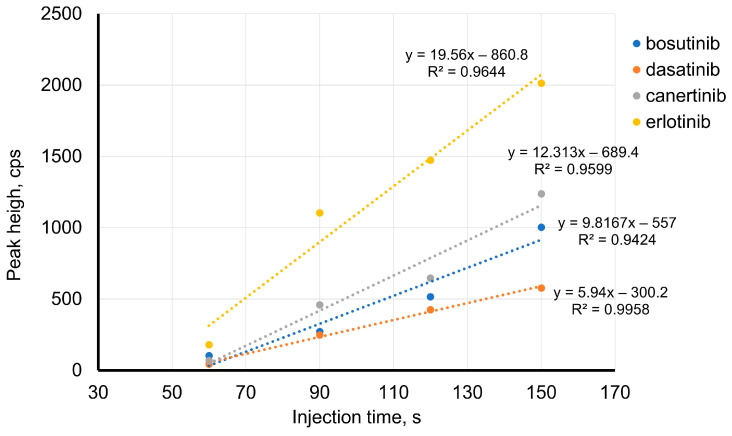
Effect of injection time on TKIs’ peak height BGE: 50 mM formic acid, pH 2.5; 120 cm-long capillary; injection pressure 50 mbar; voltage of 20 kV; concentration of TKIs: 10^−7^ mol·L^−1^.

Therefore, using a longer capillary may be the solution to improving the LOD values. If a longer capillary is used, the preconcentration ratio should be the same or better, and the TKIs should separate. Hence, a capillary of 120 cm was applied. Here, an injection time of 120 s, two-times longer than that used with an 85 cm-long capillary, represents the same portion of the capillary length (7%; 8.5 cm). With the new capillary (120 cm), the effect of the injection time of TKIs was studied in a range of 60 to 150 s (150 s represents an injection of 8.9% of the capillary length). A linear increase in the TKI peak area was observed up to 120 s for bosutinib, canertinib, and erlotinib, and up to 150 s for dasatinib. Moreover, the effect of stacking on the peak height was studied (Figure 3). The peak height linearly increased with the injection time. Hence, an injection time of 120 s was chosen for further experiments.

### 2.3. Analysis of Spiked Plasma Samples

**Figure 4:**
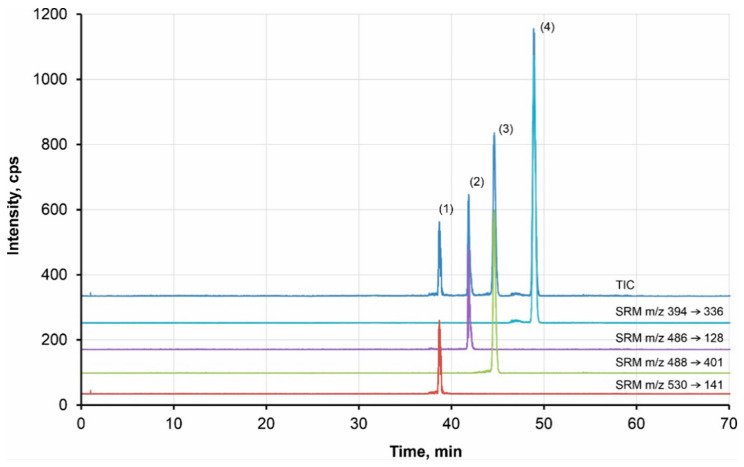
Example of analysis of the model TKIs by CE-MS/MS Peaks: (1) bosutinib, (2) canertinib, (3) dasatinib, (4) erlotinib; BGE: 50 mM formic acid, pH 2.5; 120 cm-long capillary; injection 120 s by 50 mbar; voltage of 20 kV; MS conditions: U_ESI_ = 4.0 kV, T = 250°C, 5 µL/min, 15 psi, sheath liquid: 75:24.9:0.1 MeOH, H2O, formic acid (*v*/*v*), flow rate 0.6 μL·min^−1^; SRM for quantification: bosutinib *m*/*z* 530 → *m*/*z* 141, 10 eV; dasatinib *m*/*z* 488 → *m*/*z* 401, 20 eV; erlotinib *m*/*z* 394 → *m*/*z* 336, 20 eV; canertinib *m*/*z* 486 → *m*/*z* 128, 15 eV; sample: deproteinated plasma with addition of 1·10^−7^ mol·L^−1^ TKIs.

Finally, the CE-MS/MS method was applied for the analysis of spiked plasma samples. The spiked plasma was deproteinated using trichloroacetic acid. The final sample was diluted with methanol to decrease the conductivity of the sample, allowing for the stacking preconcentration. An example of the TKI analysis at a concentration level of 10^−7^ mol·L^−1^ is given in Figure 4. It can be seen that the method allows for determination of TKIs at low concentration levels with high resolution.

Finally, the method for the determination of TKIs in plasma samples was validated in terms of linearity, LOD, LOQ, repeatability, selectivity, and recovery (Table 2). All calibrations were linear with correlation coefficients higher than 0.985. The LOD and LOQ values were in a range of 3.9–23.0 nmol·L^−1^ (1.5–11.5 ng·mL^−1^) and 11.9–69.7 nmol·L^−1^ (4.7–34.7 ng·mL^−1^), respectively. The selectivity was evaluated by comparing analyses of extracted blank plasma (without the addition of TKIs) with the analyses of TKIs at LOQ levels. There was no signal overlap. The intraday and interday repeatability (precision) of migration times varied between 0.5 and 3.2%, and 1.5 and 3.9%, respectively. The repeatability of peak heights was less than 3.2% (intraday) and 8.1% (interday). The trueness, expressed as the recovery, ranged from 96% to 103%. As can be seen in Table 2, all the data are fully acceptable for trace analysis; therefore, the method could be employed in routine therapeutic drug monitoring.

## 3. Discussion

In this work, a novel CE-MS/MS method for the determination of four model TKIs, namely bosutinib, dasatinib, canertinib, and erlotinib, was developed. The stacking online preconcentration technique was used to achieve LOD and LOQ values comparable with HPLC-MS methods, allowing for determination of all drugs at physiological levels in plasma samples. A simple extension of the capillary length (from 85 cm to 120 cm, representing a 41% increase) led to a significant decrease in LOD and LOQ values to ng·mL^−1^ levels (LODs of 1.5–11.5 ng·mL^−1^; LOQs of 4.7–34.7 ng·mL^−1^). Theoretically, the LOD and LOQ values can be improved by an extension of the capillary length. However, the increase in separation distance also led to an increase in analysis time, in some cases. In this work, the analysis lasted for 60 min; in contrast, LC-MS could finish in approx. 10 min (without column equilibration) [30,46]. This is, of course, a drawback of the CE-MS/MS method. However, this weakness is balanced by the reduced consumption of chemicals and samples by the “environmental friendliness” of CE-MS. Subsequently, LOD values were compared with a CE-MS/MS method without any preconcentration (5 s injection by 50 mbar); the preconcentration factors ranged between 12.6 (dasatinib) and 14.4 (bosutinib). Since the levels of these drugs are in ng·mL^−1^ concentrations in plasma [47,48], the method could be employed in routine therapeutic drug monitoring. In conclusion, a new CE-MS/MS method for determination of certain TKIs in plasma samples was developed and validated.

## 4. Materials and Methods

### 4.1. Chemicals and Materials

Chemicals (mainly acetic acid, formic acid, trichloroacetic acid, sodium hydroxide solution (0.1 mol·L^−1^), methanol, isopropanol, water) and standards (dasatinib, erlotinib, canertinib, and bosutinib), all of analytical grade or higher (solvents of HPLC-grade) purity, were bought from Sigma-Aldrich (St. Louis, MO, USA). Deionized water with resistivity of 18.2 MΩ.cm was prepared by the MilliQ system from Millipore (Molsheim, France).

Background electrolytes (BGEs were prepared by dissolving corresponding volumes of acids in HPLC-grade water. The pH was measured using an inoLab (WTW, Weilheim, Germany) pH meter. The ionic strength was calculated using Peakmaster software [49]. Finally, all the BGEs were filtered using 0.45 µm PTFE syringe filters (Labicom, Czech Republic).

The blood plasma sample was obtained from Sigma-Aldrich (St. Louis, MO, USA). The sample was spiked with the model TKIs and deproteinated as follows: 100 µL of the sample was mixed with 15 µL of cold trichloroacetic acid and shaken for 15 min. Then, it was centrifuged at 12,000× *g* for 5 min. Finally, the supernatant (50 µL) was carefully transferred to the sample vial for CE analysis and diluted with BGE–methanol mixture (80:20, *v*/*v*, 50 µL).

### 4.2. CE-MS

All the experiments were performed using an Agilent 7100 capillary electrophoresis instrument, which was connected to an Agilent 6460 triple quadrupole mass spectrometer (Waldbronn, Germany). The sheath liquid was delivered into the electrospray interface via an isocratic LC pump Agilent 1260 with a 1:100 flow splitter. 

Separations were performed in fused silica capillaries of 85 cm or 120 cm in length (the effective length was the same) 50 µm ID, from Molex (Lisle, IL, USA). Prior to first use, the capillaries were initially conditioned by rinsing them with 0.1 mol·L^−1^ NaOH for 20 min and then deionized water for 30 min, out of the MS. Between each sample run, the capillary was flushed with 0.1 mol·L^−1^ NaOH for 10 s, HPLC-grade water for 3 min, and BGE for 3 min. All the rinsing was carried out at a pressure drop of 935 mbar. The capillary cassette was thermostated at 25 °C, except for the part of the cassette leading to the MS interface. Each experiment was conducted in triplicate, unless stated otherwise.

### 4.3. Validation

The method was validated using the following performance characteristics: linearity, limit of detection (LOD), limit of quantification (LOQ), repeatability of migration time and peak area, and recovery. Linearity was tested using calibration within a concentration range of 1 × 10^−9^–1 × 10^−5^ mol·L^−1^ for all the TKIs. LODs and LOQs were calculated according to equations: LOD = 3.3 SD/s and LOQ = 10 SD/s, where SD is the standard deviation of the signal intensity and s is the slope of the calibration curve. The selectivity was investigated by comparing analyses of blank plasma without the addition of TKIs with the analyses of TKIs at LOQ levels. Acceptance criteria for any interference included a signal response lower than 5% of the LOQ. The reproducibility of migration times and peak areas was calculated from repeated analyses at the 1 × 10^−7^ mol·L^−1^ level; the intraday repeatability was calculated from three repetitions within one day; and the interday repeatability was calculated from repetitive analyses on three consecutive days (with three repetitions each day). The recovery was evaluated using analyses of TKIs in blood samples spiked at the 1 × 10^−7^ mol·L^−1^ level; the recovery was calculated as the ratio of the determined TKI concentration and the true (added) concentration.

## 5. Conclusions

In this work, a novel CE-MS/MS method was employed for the determination of four model TKIs, namely bosutinib, dasatinib, canertinib, and erlotinib. The stacking online preconcentration technique was used with a 120 cm-long capillary to achieve LOD and LOQ values comparable with HPLC-MS methods, allowing for determination of all drugs at physiological levels in plasma samples. Under optimal conditions, 50 mM formic acid pH 2.5, an injection time of 120 s, and an optimized mass spectrometry set-up (sheath liquid composition 75:24.9:0.1 (*v*/*v*) methanol, water, formic acid, and appropriate conditions for ion transitions), LODs in a range of 3.9–23.0 nmol·L^−1^ were observed.

## Figures and Tables

**Table 1:** SRM transitions of model TKIs.

Compound	Parent Ion (*m*/*z*)	Quantification Transition Ion (*m*/*z*)	Collision Energy for Quantification Transition (eV)	Confirmation Transition Ion (*m*/*z*)	Collision Energy for Confirmation Transition (eV)
Dasatinib	488	401	20	232	40
Erlotinib	394	336	20	278	30
Canertinib	486	128	15	100	40
Bosutinib	530	141	10	113	50

**Table 2:** Summary of the method validation.

Parameter	Analyte			
	Bosutinib	Dasatinib	Canertinib	Erlotinib
Calibration range (mol × L^−1^)	1 × 10^−8^–1 × 10^−5^	3×10^−9^–1 × 10^−5^	3×10^−9^–1 × 10^−5^	3×10^−9^–1 × 10^−5^
Calibration equation	y = 1.528 × 10^8^x + 145	y = 1.148 × 10^8^x + 94	y = 4.977 × 10^8^x + 167	y = 8.417 × 10^8^x + 385
Correlation coefficient	0.9921	0.9849	0.9976	0.9943
LOD (nmol·L^−1^)	21.6	23.0	8.0	3.9
LOQ (nmol·L^−1^)	65.4	69.7	24.1	11.9
Intraday repeatability of migration time (%)	0.55	2.11	1.01	3.18
Interday repeatability of migration time (%)	1.76	3.76	1.57	3.85
Intraday repeatability of peak heights (%)	3.17	1.94	2.62	2.20
Interday repeatability of peak heights (%)	5.89	5.21	8.11	3.60
Recovery (%)	103.2	96.4	96.1	101.5

## Data Availability

The data are available from the author upon reasonable request.
